# The Asgard Archaeal-Unique Contribution to Protein Families of the Eukaryotic Common Ancestor Was 0.3%

**DOI:** 10.1093/gbe/evab085

**Published:** 2021-04-23

**Authors:** Michael Knopp, Simon Stockhorst, Mark van der Giezen, Sriram G Garg, Sven B Gould

**Affiliations:** 1 Institute for Molecular Evolution, Heinrich-Heine-University Düsseldorf, Germany; 2 Centre for Organelle Research, University of Stavanger, Norway

**Keywords:** eukaryote origin, eukaryogenesis, asgard archaea, compartmentalization

## Abstract

The identification of the asgard archaea has fueled speculations regarding the nature of the archaeal host in eukaryogenesis and its level of complexity prior to endosymbiosis. Here, we analyzed the coding capacity of 150 eukaryotes, 1,000 bacteria, and 226 archaea, including the only cultured member of the asgard archaea. Clustering methods that consistently recover endosymbiotic contributions to eukaryotic genomes recover an asgard archaeal-unique contribution of a mere 0.3% to protein families present in the last eukaryotic common ancestor, while simultaneously suggesting that this group’s diversity rivals that of all other archaea combined. The number of homologs shared exclusively between asgard archaea and eukaryotes is only 27 on average. This tiny asgard archaeal-unique contribution to the root of eukaryotic protein families questions claims that archaea evolved complexity prior to eukaryogenesis. Genomic and cellular complexity remains a eukaryote-specific feature and is best understood as the archaeal host’s solution to housing an endosymbiont.

Significance

Ever since the first report of a new archaeal lineage, the asgard archaea, their metagenome analyses have encouraged continued speculations on a type of cell biology ranging between that of prokaryotes and eukaryotes. Although it appears a tempting notion, recent microscopic images of an asgard archaeon suggest otherwise. We inspected the origins of eukaryotic protein families with respect to their distribution across bacteria and archaea. This reveals that the protein families shared exclusively between asgard archaea and eukaryotes amounts to only 0.3% of the protein families conserved across all eukaryotes. Asgard archaeal diversity is likely unrivaled across archaea, but their cell biology remains prokaryotic in nature and lends support for the importance of endosymbiosis in evolving eukaryotic traits.

## Introduction

Four billion years of prokaryotic evolution has only once resulted in the emergence of highly compartmentalized cells and eventually macroscopic body plans: following the origin of eukaryotes through endosymbiosis. The difference between pro- and eukaryotic biology is evident and the lack of intermediates between the two types of cells places endosymbiosis at the event horizon of eukaryogenesis. The analysis of core eukaryotic features such as the nucleus, mitochondria, sex and meiosis, compartmentalization and dynamic membrane trafficking, and virtually all of the associated protein families, consistently point to their presence in the last eukaryotic common ancestor (LECA) (Fritz-Laylin et al. 2010; Koonin et al. 2013; Koumandou et al. 2013; Garg and Martin 2016). We possess a reasonable understanding of the basic cellular features and coding capacity of LECA, owing to the growing number of genome sequences spanning all of eukaryotic diversity. All eukaryotes stem from a single ancestor that in terms of cellular and genomic complexity rivaled those of extant eukaryotic supergroups (Fritz-Laylin et al. 2010; Koonin et al. 2013; Koumandou et al. 2013). There is general consensus that LECA was a product of the integration of an alphaproteobacterium into an archaeal host following endosymbiosis (Lane 2011; Blackstone 2013; Martin et al. 2015; Dacks et al. 2016; Spang et al. 2019).

Through the description of the asgard archaea, current debates once again concern the cellular complexity of the host that came to house the endosymbiont and what contribution the mitochondrion could have played in establishing the eukaryotic cell (Dacks et al. 2016; Martin et al. 2017). Asgard archaea, a novel phylum assembled from metagenome data, are viewed as bridging the gap between pro- and eukaryotic cells, because they encode proteins homologous to eukaryotic ones that are involved in intracellular vesicle trafficking and the regulation of actin cytoskeleton dynamics (Spang et al. 2015; Zaremba-Niedzwiedzka et al. 2017; Neveu et al. 2020). The cellular complexity of the host cell that acquired the alphaproteobacterial endosymbiont has been a matter of speculation ever since the realization that endosymbiosis was pivotal in the transition to eukaryotic life. Modern models of eukaryogenesis differ regarding the timing of mitochondrial acquisition, the extent of the cellular complexity of the host, and the selective reasons provided for explaining the presence, function, and emergence of eukaryotic traits prior or ensuing endosymbiosis (O’Malley 2010; Martin et al. 2015; Gould et al. 2016; Tria et al. 2021; Vosseberg et al. 2020).

Understanding the steps of eukaryogenesis is a demanding intellectual challenge that explores the past of life and one of its most radical transitions. It holds the key to understanding the steps toward cellular complexity, the timing of mitochondrial entry, and what limits prokaryotes to frequently evolve eukaryote-like complexity. Was eukaryogenesis really a matter of luck (Booth and Doolittle 2015) and how important was the benefit provided by the mitochondrion to the host cell (Lane and Martin 2010; Lynch and Marinov 2015; Lane and Martin 2016) or the possible availability of altering terminal electron acceptors (Speijer 2017)? Any model that views endosymbiosis as some kind of terminal coincidence on the evolutionary roadmap to the eukaryotic domain of life needs to explain the singularity that is eukaryogenesis and the lack of comparable complexity among prokaryotes.

A consistent motivation for speculating on the archaeal host cell’s grade of complexity is trying to understand whether the host cell was phagocytotic or not (Cavalier-Smith 1987; Yutin et al. 2009; Martijn and Ettema 2013; Martin et al. 2017), and one that would offer an explanation for the mode of endosymbiont entry. This is complicated by the description of a phagocytosis-like process in a planctomycete (Shiratori et al. 2019) and the conflicting evidence for intracellular prokaryotic endosymbionts in the absence of phagocytosis (Embley and Finlay 1993; Fenchel and Bernard 1993; Schmid 2003; Duplessis et al. 2004; Zientz et al. 2004; Thacker 2005; Woyke et al. 2006; Husnik et al. 2013). Some of the asgard archaea encode actin-regulating profilins (Akıl and Robinson 2018), small Rab-like GTPases (Surkont and Pereira-Leal 2016), and prototypic SNARE proteins (Neveu et al. 2020), but they are not phagocytotic (Burns et al. 2018). Phagocytosis might have evolved multiple times independently (Yutin et al. 2009; Mills 2020) and is a mode of feeding, which is incompatible with the syntrophic foundation that underpins eukaryogenesis (Martin and Müller 1998; Vellai et al. 1998; Martin et al. 2015; Spang et al. 2019; Imachi et al. 2020). A sole focus on this single eukaryotic trait might distract and furthermore discounts the complexity of the transition that was involved. What is certain is that images of an asgard archaeon, Candidatus *Prometheoarchaeum syntrophicum* MK-D1, reveal cells with typical archaeal morphology, half a micron in diameter, with obligate syntrophy, and devoid of any endomembrane system (Imachi et al. 2020).

Here, we clustered the available genomes of 150 eukaryotes, 1,000 bacteria, and 226 archaea (including asgard archaea metagenomic assemblies, and for comparison the complete genome of the cultured Candidatus *P. syntrophicum* strain MK-D1) in order to evaluate the asgard archaeal-unique contribution to eukaryogenesis that is understood as support for early cellular complexity in asgard archaea.

## Materials and Methods

### Calculation of Protein Families

Prokaryotic gene families were calculated from complete genomes of 1,000 bacteria and 226 archaea of the RefSeq database (version September 2016) including 11 representatives of the asgard archaea (Zaremba-Niedzwiedzka et al. 2017) and Candidatus *P. syntrophicum* MK-D1 (Imachi et al. 2020), separately. Bacterial and archaeal protein families were calculated via MCL (van Dongen 2000; Enright 2002) (–abc -scheme 7) from all reciprocal best BLAST hits with pairwise global identities (Rice et al. 2000) of at least 25% identity and a maximum *e*-value of 1 × 10^−10^ among all investigated bacteria and archaea, respectively. Eukaryotic gene families on the basis of 150 eukaryotic genomes were calculated as part of a previous study (Brueckner and Martin 2020). Prokaryotic and eukaryotic clusters were combined into EPCs if at least 50% of all sequences of a eukaryotic cluster had their best hit in a prokaryotic cluster and vice versa according to the “reciprocal best cluster approach” described in Ku et al. (2015). All proteomes used in this study are listed in supplementary table 5 (Supplementary Material online). Protein families of Methanococci and Crenarchaeota were calculated from all pairwise global identities of all protein sequences, using the above-mentioned identity and *e*-value cutoffs, via MCL (van Dongen 2000; Enright 2002).

### BLAST Hit Analysis

We performed a Diamond BLASTp (Buchfink et al. 2015) hit analysis on all protein sequences of the 12 asgard archaeal proteomes including MK-D1. We compared the results with 12 bacterial and archaeal proteomes each, plus eight eukaryotic ones. We blasted all protein sequences of the chosen proteomes against our database of 5655 prokaryotic and 150 eukaryotic proteomes. For each proteome, we quantified the number of sequences that showed significant hits (at least 25% identity and a minimal *e*-value of 1 × 10^−5^) within bacteria, archaea, eukaryote, or any combination of these three. To counter overrepresentation of some genera within our database, we excluded hits of the same genus for all tested protein sequences.

### InterProScan ESP Analysis

InterProScan version 5.39-77.0 (Quevillon et al. 2005) with standard parameters was used to annotate all asgard archaeal proteomes, the MK-D1 proteome and all 214 archaeal proteomes within our prokaryotic database. As in Zaremba-Niedzwiedzka et al. (2017), we searched for InterPro-Identifiers that correspond with Eukaryote-specific proteins and plotted the results of all investigated asgard archaea together with the results of 14 model RefSeq archaea for comparison (see supplementary fig. 3, Supplementary Material online).

### LECA Cluster Filtering

Because eukaryotic inheritance is strictly vertical, the eukaryotic protein families were filtered for families that contained at least one protein sequence from one member of each of the six supergroups included in our data set of 150 eukaryotes, being *Archaeplastida*, *Opisthokonta*, *SAR*, *Hacrobia*, *Excavata*, and *Mycetozoa* resulting in 1,880 protein families passing this criterion.

## Results

In order to evaluate to what degree asgard archaea bridge the prokaryotic and eukaryotic protein families, we performed a global comparison of clustered gene families across 11 asgard archaeal metagenome-assembled genomes (MAGs), the closed MAG of the cultured asgard archaeon Candidatus *P. syntrophicum* MK-D1, 214 other archaea, 1,000 bacteria, and 150 eukaryotes. Protein families for 150 eukaryotes were taken from Brueckner and Martin (2020), which included 239,012 clusters. We further clustered proteins from the prokaryotes, resulting in 352,384 bacterial clusters and 49,855 archaeal clusters. Subsequently, the eukaryote and prokaryote clusters (EPCs) were merged in a reciprocal best cluster approach previously described in Ku et al. (2015), yielding EPCs. These EPCs contained proteins from eukaryotes and proteins from either archaea (eukaryote-archaea clusters, EA) or bacteria (eukaryote-bacteria clusters, EB), or both (eukaryote-archaea-bacteria clusters, EAB). This approach yielded 2,590 EPCs, of which 867 or 33.5% (330 EA clusters + 537 EAB clusters; supplementary table 1, Supplementary Material online) contained an archaeal component. Among these 867 EPCs, asgard archaeal protein sequences, including those of Candidatus *P. syntrophicum*, were present in about 75% of the protein families (75% of EAB clusters and 75.2% of EA clusters).

**Fig. 1.**
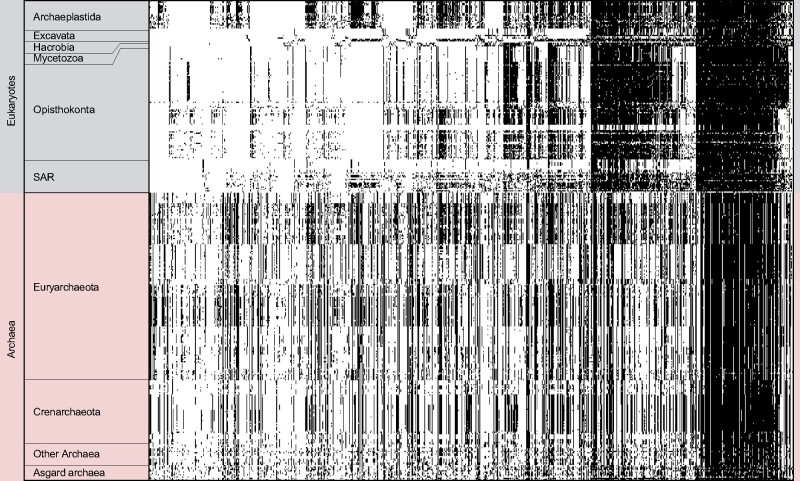
PAP of all 867 EPCs with archaeal contribution among the investigated 150 eukaryotes, 214 archaea and 12 asgard archaea. The protein families were sorted by their distribution among six eukaryotic supergroups (SAR; Stramenopila, Alveolata, Rhizaria), their presence is indicated in black along the X axis. The group of “Other Archaea” comprised all archaeal groups within our database that have less than 15 members. The distribution of these EPCs among the asgard archaea does not reveal a particular pattern such as that seen for the other archaeal groups, demonstrating their large genetic differences.

A presence-absence pattern (PAP) of all 867 protein families with archaeal contribution to the EPCs, including the 537 protein families present in all domains is shown in figure 1 (and supplementary table 1, Supplementary Material online). Although gene distributions among eury- and crenarchaeota is highly similar, those of the asgard archaea are patchier and more diverse. Among all of our 239,012 eukaryotic clusters, we could identify only six EA clusters with asgard archaeal-unique contributions to eukaryotes (supplementary table 2, Supplementary Material online), representing 0.0025% of all extant eukaryotic diversity. To calculate the asgard archaeal-unique contribution to LECA, we filtered the eukaryotic clusters for those that include at least one representative of each of the six eukaryotic supergroups, resulting in 1880 LECA clusters and consequently an asgard archaeal-unique contribution of 0.3191%.

**Fig. 2:**
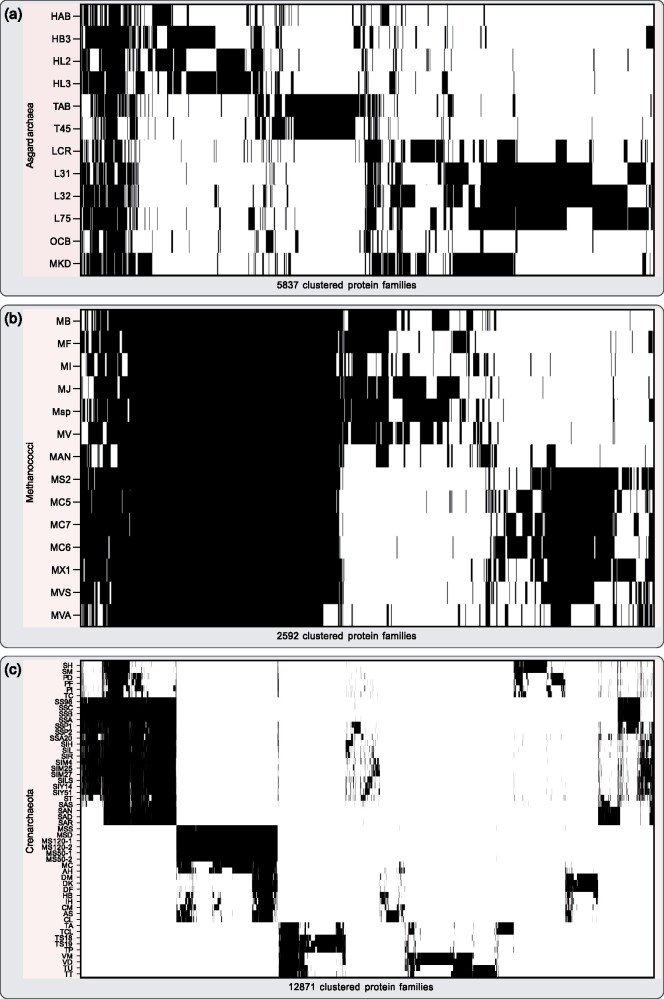
Archaeal protein family distributions. (*a*) Distribution of all 5,837 calculated asgard archaeal protein families among the investigated asgard archaea. The protein families were obtained by globally comparing all asgard archaeal protein sequences in a pairwise all-versus-all Diamond BLASTp approach including subsequent clustering via MCL. The result was sorted via hierarchical clustering along the *X* axis. The vast majority of protein families is not shared among all asgard archaea but they are rather specific to the individual taxon. Candidatus Prometheoarchaeon syntrophicum MK-D1 shares the highest number of its protein families with members of the Lokiarchaeota. (*b*) For comparison, we calculated protein families for 14 members of the class Methanococci in the same manner, generating a total of 2,592 protein families. Hierarchical clustering revealed a striking difference in protein families shared between members within the two clusterings, underlining the investigated asgard archaea’s diversity among each other. (*c*) On the contrary, clustering of 52 members of the crenarchaeota, a highly diverse taxonomic group, results in 12,871 protein families. The matrix was sorted along the X and Y axis via hierarchical clustering. All Abbreviations used are listed in supplementary table 6, Supplementary Material online.

But how closely are the asgard archaea related to each other with respect to other archaea? To investigate their kinship, we constructed protein families of only the asgard archaeal protein sequences, which generated a set of 5,837 protein families. The distribution of these protein families among the asgard archaea reveals a pronounced diversity, with only a small proportion of the protein families being shared across all of them, which suggests one is dealing with a kind of superphylum (fig. 2*a*). This is evident from a comparison wherein we clustered all protein sequences of 14 members of the genus Methanococci and 52 crenarchaeote members from the TACK superphylum (fig. 2*b* and c), revealing 2,592 and 12,871 protein families, respectively.

A key difference between archaea and eukaryotes is the difference in the number of protein families. In a previous study, 239,012 protein families were generated on the basis of all pairwise protein sequence comparisons of 150 eukaryotic proteomes (Brueckner and Martin 2020). The same approach yields a total of 49,855 protein families on the basis of all pairwise protein sequence comparisons between 226 archaea, including 11 asgard archaea and Candidatus *P. syntrophicum* MK-D1. The patchy distribution of the asgard archaeal EPCs (fig. 1) and the high diversity between each other (fig. 2*a*) prompts a closer look, but the vast difference in the number of protein families reflects the sudden inflation of protein family emergence at the origin of eukaryotes.

**Fig. 3:**
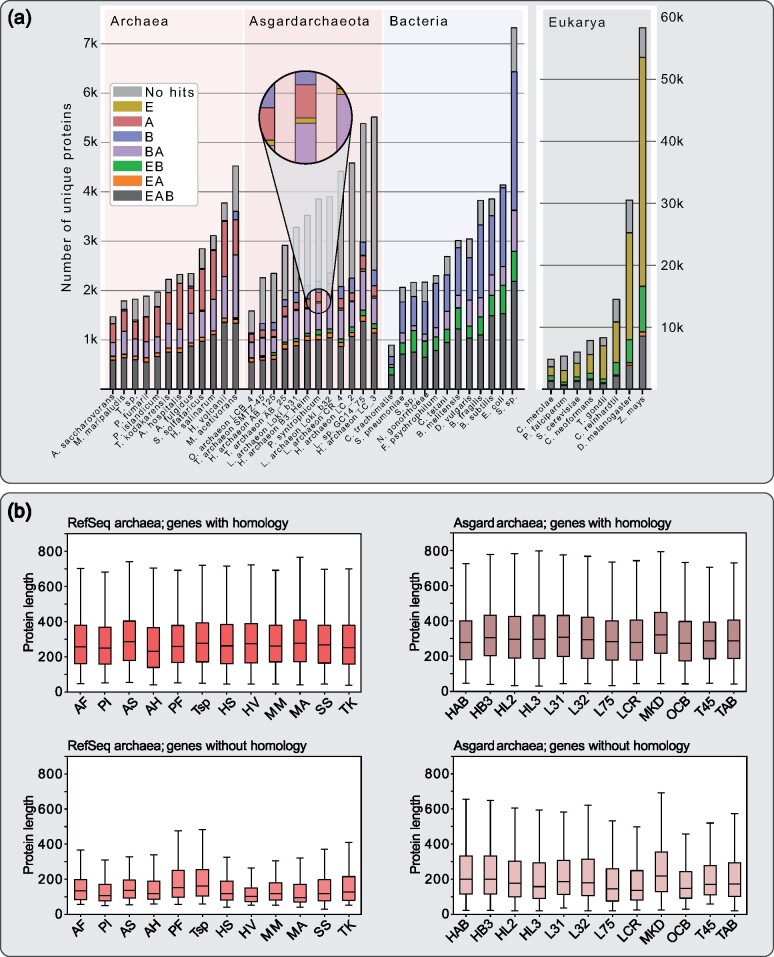
Distribution of protein homologs and length comparison of archaeal genes with and without homology. (*a*) BLAST analysis comparing the proteomes of known archaea and bacteria as well as the twelve investigated asgard archaea. Sequences were blasted against a prokaryotic database comprising 5,655 prokaryotic genomes (including the 1,000 bacteria and 212 of the archaea which were used for protein family calculation) and a eukaryotic database comprised of 150 genomes. For each organism, we quantified the number of sequences with subjects among eukaryotes, bacteria or archaea only, or any combination of these three. To counter possible biases in database composition, hits from the same genus were excluded. The close up highlights one exemplary case of asgard archaeal proteins with hits only in eukaryotes (hence mustard colored), which represent the asgard archaeal-unique contribution to eukaryotic protein families. E, eukaryotes; A, archaea; B, bacteria; AB, archaea-bacteria, etc. (*b*) For proteins with- or without database hits, protein length distributions are shown as box and whiskers plots. This reveals a noticeable difference in protein lengths of sequences without database hits from only the asgard archaea compared with their sequences with database hits, and in comparison, to those of RefSeq archaea. *P* values of FDR-corrected, pairwise double-sided Kolmogorov–Smirnov tests in supplementary table 7, Supplementary Material online. All used abbreviations are listed in supplementary table 6, Supplementary Material online.

Because many asgard archaeal protein sequences could not be clustered due to a lack of similar sequences that would represent homologs, we conducted an analysis of BLASTp hits against a bacterial database consisting of 5,443 proteomes, an archaeal database consisting of 212 proteomes (excluding Asgard archaea and Hadesarchaea) and a eukaryotic database consisting of 150 proteomes. For all investigated asgard archaea and a selection of 12 well-known and diverse archaea and bacteria each, as well as eight eukaryotes, we quantified the amount of sequences with homologs in one of these databases, any combination of these, or no significant homologs at all (fig. 3*a*), whereas ignoring hits from the same genus to counter database composition biases. For asgard archaea, only 27 protein sequences on average have homologs unique to eukaryotes (fig 3*a*, magnified area), supporting the initial EPC analysis which only uncovered six protein families that were unique to eukaryotes and asgard archaea. Furthermore, this test reveals that high proportions of the asgard archaeal proteomes do not retrieve significant hits in our three tested databases (fig. 3*a*, gray bars). In a few cases, such as for example for Candidatus *Heimdallarchaeota LC_3* or Candidatus *Lokiarchaeota archaeon CR_4*, the number of proteins for which no homology was detected in any other species, exceeds half of the respective genome’s coding capacity (fig. 3*a*).

We plotted the protein sequence length distributions for each proteome, separating hits with and hits without significant database hits (fig. 3*b*). Although the length distributions of sequences with significant database hits were comparable between the asgard- and the RefSeq archaea, the distributions of hits without significant database hits show a major difference (fig. 3*b*). Furthermore, simulating missing data by ignoring not only hits from the same genus but also the same phylum produced a more similar result for the reference organisms and asgard archaea (supplementary fig. 1, Supplementary Material online), recapitulating the extensive diversity among the latter.

## Discussion

The identification of asgard archaea from deep-sediment metagenome data (Seitz et al. 2016; Zaremba-Niedzwiedzka et al. 2017) provides valuable new information from which to re-evaluate key issues surrounding the tree of life and the emergence of its eukaryotic branch. To begin with, the iconic tree that introduced three aboriginal lineages (Woese and Fox 1977) might require a revision. Phylogenomic analysis of asgard archaea provides evidence for a two-domains tree and the emergence of the host cell lineage of eukaryogenesis from within the archaeal domain (Cox et al. 2008; McInerney et al. 2014; Hug et al. 2016; Williams et al. 2020) with some skepticism, however, remaining (Forterre 2015; Da Cunha et al. 2018; Liu et al. 2021). Parallel to the discovery of the asgard archaea were immediate speculations regarding their cellular complexity (Dacks et al. 2016; Pittis and Gabaldón 2016; Rout and Field 2017; Akıl and Robinson 2018; Zachar et al. 2018; Neveu et al. 2020) and a faith of having identified the missing link between pro- and eukaryotic biology based on the identification of a few eukaryote signature proteins (ESPs), which we find to be 27 on average. In light of these numbers, the potential of these archaea to display eukaryote-like cell complexity is hard to maintain.

Our analysis confirms a patchy distribution of ESPs among asgard archaea (Dacks et al. 2016; Klinger et al. 2016; Zaremba-Niedzwiedzka et al. 2017; Bulzu et al. 2019; Imachi et al. 2020; Inoue et al. 2020; Liu et al. 2021) (fig. 1). Although of course the archaeal host brought in 1,000s of genes, the unique contribution of this lineage to the eukaryotic protein families is substantially less than what one might infer from the original metagenome reports and subsequent interpretations (Dacks et al. 2016; Pittis and Gabaldón 2016; Rout and Field 2017; Akıl and Robinson 2018; Zachar et al. 2018; Neveu et al. 2020; Vosseberg et al. 2020). The irregular gene distribution (fig. 2*a*) might reflect differential gene loss upon the segregation of the common ancestor of asgard archaea and the archaeal host cell lineage (Eme et al. 2017). Considering the role of pangenomes in the transformation of prokaryotic lineages and the conquering of ecological niches (McInerney et al. 2017), pangenomes offer a complementary explanation to the differential loss of genes. The archaeal ancestor of eukaryotes might have tapped a gene pool more extensively than the sister lineages leading to extant asgard archaea.

The notion that asgard archaeal contributions to eukaryotes were higher only to be eventually replaced by bacterial (endosymbiotic) contributions might be brought up (Pittis and Gabaldón 2016; Eme et al. 2017; Vosseberg et al. 2020). The absence of extant archaeal relatives with similar distributions of ESPs indicates that archaea neither have the necessity nor the selective pressure for maintaining ESPs in the absence of an endosymbiont. Any theory that hinges upon a larger presence of ESPs in archaea (or bacteria) prior to mitochondrial endosymbiosis ignores the complete lack of such an accumulated number of ESPs in extant prokaryotes to a degree that even remotely matches that of any given eukaryotic lineage. The absence of ESPs in the archaeal host lineage could be attributed to the combined effect of selection and extinction, rendering the unique combination of ESPs of such a hypothetical host lineage only advantageous for a brief period of time. Although feasible, the acknowledgment of such mechanisms in evolution then also applies to the endosymbiont. For the mitochondrion, however, the presence of bacterial genes that do not clearly branch with alphaproteobacteria are often interpreted as LGT events (Pittis and Gabaldón 2016; Eme et al. 2017; Vosseberg et al. 2020). Any considerations on the environmental conditions and evolutionary pressures that promoted the evolution and origin of ESPs, must also be equally considered for any genes that are currently thought to be recent independent gene transfer events from prokaryotes to eukaryotes and not of endosymbiotic (i.e., alphaproteobacterial) origin.

Our protein family clustering method, which readily detects the mitochondrial contribution (and the cyanobacterial contributions in the case of the Archaeplastida; supplementary fig. 2, Supplementary Material online) failed to detect a comparable asgard archaeal-unique contribution. A stacked bar diagram puts gene family cluster contribution in each lineage into a global perspective (fig. 3*a*; supplementary fig 1, Supplementary Material online). There is a small proportion of eukaryotic homologs (E, mustard yellow) visible, for example, in the Candidatus *P. syntrophicum* MK-D1, but it is substantially smaller in comparison to the eukaryote-bacteria (EB, green) specific homologs evident in eukaryotes (and bacteria vice versa) that reflects the mitochondrial contribution to eukaryogenesis (Brueckner and Martin 2020). Neglecting the surprisingly low number of asgard archaeal-unique homologs to eukaryotic genomes, our analysis demonstrates that asgard archaea are among the most genetically diverse group of archaea when comparing it to the genus Methanococci and the phylum Crenarchaeota (fig. 2). The odd length distributions of the asgard archaeal proteins with no homology (fig. 3*b*) are strange as well, because protein length across pro- and eukaryotes is usually well conserved (Xu et al. 2006). This could hint at an assembly and/or binning issue, which was also observed regarding the anomalous phylogenetic behavior of their ribosomal proteins and concatenated gene trees (Da Cunha et al. 2018; Garg et al. 2021). If not, it is a biological phenomenon absent in other sequenced prokaryotes that requires explaining.

Considering the amount of data gathered in just a few years (Klinger et al. 2016; Villanueva et al. 2017; Bulzu et al. 2019; Imachi et al. 2020; Inoue et al. 2020; Liu et al. 2021), it is surprising asgard archaea have escaped identification for so long. Their habitats had been sampled before, so it is likely that the method used and maybe an obligate dependency on syntrophy hindered culturing, except for one imposing exception (Imachi et al. 2020). Dedicated phylogenomic efforts are necessary to resolve their taxonomic classification, whereas only culturing can picture their cell morphology.

Analyses of asgard archaeal ESPs show they have the potential to function similar to their eukaryotic homologs in a eukaryotic system (Klinger et al. 2016; Rout and Field 2017; Akıl and Robinson 2018; Neveu et al. 2020), but cross-kingdom inferences have their limits (Dey et al. 2016). The analysis of archaeal small GTPases (Surkont and Pereira-Leal 2016) and homologs of ESCRT proteins, the CDVs (Lindås et al. 2008; Lindås and Bernander 2013; Caspi and Dekker 2018), serve as examples. One needs to interpret asgard archaeal ESPs in their prokaryotic context and in cells lacking an endosymbiont. The images of an asgardarchaeon, those of Candidatus *P. syntrophicum* MK-D1 and its dependency on a bacterial partner (Imachi et al. 2020), define the current standard from which to plot eukaryogenesis and the steps leading to eukaryotic cell and genome complexity.

The identification of the asgard archaea and the culturing of one representative represent an important milestone in micro- and evolutionary biology. Their phylogenetic analysis echoes two previously predicted outcomes: 1) eukaryotes to branch from within archaea, solidifying the two-domains tree of life, and 2) that the closer we zoom in on the two prokaryotic partners from which eukaryotes evolved, the higher the number of otherwise eukaryote-typical genes we identify in prokaryotes. The description of Candidatus *P. syntrophicum* MK-D1 (Imachi et al. 2020) reminds us to not conflate genotypic with phenotypic complexity. This predicts that future asgard archaea we see cultured will lack eukaryotic traits, too, and most, if not all, will depend on syntrophy. Our analysis also predicts that much of asgard archaeal diversity remains to be described and will require further taxonomic sorting, but that the gap between the pro- and eukaryotic protein families will remain decisive and to change little. Placing the endosymbiotic event and the energetic benefit offered by mitochondria to fuel the transition early in eukaryogenesis, explains the lack of physical evidence for eukaryote-like complexity in asgard archaea despite them encoding ESPs. It offers a comprehensive full-service theory for the singularity that is the origin of the eukaryotic cell that mitochondria-late models fail to provide.

## In Memoriam

During the revision of this manuscript, we learned with great sadness of the passing of Tom Cavalier-Smith, who made important contributions to the field of eukaryotic evolution and who will be missed by many.

## Supplementary Material

Supplementary data are available at *Genome Biology and Evolution* online.

## Supplementary Material

evab085_Supplementary_Data
